# Hyaluronic Acid Concentration in Pleural Fluid: Diagnostic Aid for Tuberculous Pleurisy

**DOI:** 10.14740/jocmr1980w

**Published:** 2014-10-16

**Authors:** Yusuke Yoshino, Yoshitaka Wakabayashi, Kazunori Seo, Ichiro Koga, Takatoshi Kitazawa, Yasuo Ota

**Affiliations:** a Department of Internal Medicine, Teikyo University School of Medicine, 2-11-1 Kaga, Itabashi-ku, Tokyo 173-8606, Japan

**Keywords:** Hyaluronic acid, Adenosine deaminase, Tuberculosis pleurisy, Diagnostic aid

## Abstract

**Background:**

A high concentration of hyaluronic acid in pleural fluid is suggestive of malignant mesothelioma. However, a relatively high concentration of hyaluronic acid was also seen in the pleural fluid of patients with benign inflammatory diseases. To show the utility of measuring hyaluronic acid levels in pleural fluid to diagnose tuberculous pleurisy, we compared the clinical features and levels of hyaluronic acid in the pleural fluid of patients with and without tuberculous pleurisy.

**Methods:**

We enrolled 27 patients with infective pleurisy admitted at Teikyo University Hospital from January 2010 to December 2013. Ten patients were diagnosed with tuberculous pleurisy, and 17 with non-tuberculous pleurisy. We reviewed the clinical features and data of all 27 patients and compared the two groups. We analyzed and compared the concentration of hyaluronic acid and adenosine deaminase in their pleural fluid.

**Results:**

Patients with tuberculous pleurisy tended to have significantly higher concentrations of hyaluronic acid and adenosine deaminase in their pleural fluid (tuberculous pleurisy patients vs. other infective pleurisy patients: hyaluronic acid (× 10^3^ ng/mL); 42.9 ± 23.3 vs. 16.8 ± 17.9, P = 0.003, adenosine deaminase (IU/L); 89.7 ± 33.3 vs. 74.0 ± 90.9, P = 0.032). Receiver operating characteristic analysis revealed no significant difference in the area under the curve of hyaluronic acid and adenosine deaminase volumes in pleural fluid, suggesting their equivalent value as major diagnostic tools for tuberculosis pleurisy.

**Conclusions:**

Hyaluronic acid concentration in pleural fluid can be a valuable tool for the diagnosis of tuberculous pleurisy.

## Introduction

Hyaluronic acid is an anionic, non-sulfated glycosaminoglycan distributed widely throughout connective, epithelial, and neural tissues [1]. In pleural fluid, it appears to decrease the friction between the lung and the thoracic cavity [1].

A high concentration of hyaluronic acid (> 100 µg/mL) in pleural fluid is suggestive of malignant mesothelioma [2]. In addition, a previous study showed a relatively high concentration of hyaluronic acid in the pleural fluid of patients with benign inflammatory diseases, such as rheumatoid arthritis and complicated parapneumonic effusion [3, 4]. Hyaluronic acid in pleural fluid was suggested to contribute to the early identification of complicated parapneumonic effusion [4].

Tuberculous pleurisy is one of the major manifestations of a tuberculosis infection. It is difficult to diagnose because the positive culture rate for tuberculosis in pleural fluid is usually low [5]. Therefore, measuring adenosine deaminase in pleural fluid is currently the most valuable tool for its diagnosis.

In this study, we analyzed the volume of hyaluronic acid in pleural fluid in patients of tuberculous pleurisy, to determine whether hyaluronic acid can aid in the diagnosis of tuberculous pleurisy.

## Methods

### Study population

In all, 133 patients (> 18 years) with infective pleurisy that were enrolled for the study were admitted to the Teikyo University Hospital, a 1,200-bed teaching hospital in Tokyo, Japan, from January 2010 to December 2013.

### Case definition and inclusion/exclusion criteria

Infective pleurisy was diagnosed on the basis of a positive result on culture of pleural fluid and signs and symptoms of pleurisy, such as pleural fluid, fever, chest pain, cough, fatigue, disorientation, hypotension, and respiratory failure.

Patients whose pleural fluid was collected for the analysis of adenosine deaminase, lactate dehydrogenase, and hyaluronic acid were included. In contrast, patients who had a history of respiratory malignancy and rheumatoid arthritis were excluded because previous reports have shown that respiratory malignancy including malignant mesothelioma could increase hyaluronic acid in pleural fluid [2].

### Endpoint

The endpoint of this study was to show whether hyaluronic acid in pleural fluid was significantly increased in patients with tuberculous pleurisy. In order to assess the diagnostic value of hyaluronic acid concentration in pleural fluid, it was compared with the level of adenosine deaminase.

### Statistical analysis

The results are expressed as mean with standard deviation unless otherwise indicated. For univariate analysis, Student’s *t*-test and Fisher’s exact test were used to analyze continuous and categorical data where appropriate.

To assess and compare the diagnostic accuracy of hyaluronic acid and adenosine deaminase for discriminating patients with pleurisy from those with tuberculous pleurisy, we plotted receiver operating characteristic (ROC) curves and calculated the areas under the curves (AUC) for comparison. ROC curves were generated by plotting the relationship of the true positivity (sensitivity) and the false positivity (1 - specificity) at various cut-off points in the tests.

All P-values were two-sided, and were considered statistically significant at a value of < 0.05.

### Ethics

The Ethics Committee of the University of Teikyo approved this project.

## Results

Only 33 of 133 met the inclusion criteria. Of them, six patients had histories of respiratory malignant diseases and were excluded. Twenty-seven patients were eventually analyzed: 10 had tuberculous pleurisy and 17 had non-tuberculous infective pleurisy.

We summarized demographic data, clinical features, and pleural fluid laboratory data of all 27 patients in Table 1. There were significant differences in age and levels of hyaluronic acid, and adenosine deaminase in pleural fluid between the two groups.

**Table 1:** Clinical Data for Pleurisy Patients Included in the Study Population

	All (n = 27)	Tuberculosis (n = 10)	Non-tuberculosis (n = 17)	P
Gender (male)	21 (77.8%)	8 (80.0%)	13 (76.5%)	1.00
Age (years)	70.1 ± 19.1	57.1 ± 20.6	79.7 ± 11.5	0.032
Comorbidity				
Liver cirrhosis	5 (18.5%)	1 (10.0%)	4 (23.5%)	0.621
Diabetes mellitus	8 (29.6%)	3 (30.0%)	5 (29.4%)	1.00
Chronic respiratory diseases	2 (7.41%)	1 (10.0%)	1 (5.88%)	1.00
Chronic heart diseases	7 (25.9%)	2 (20.0%)	5 (29.4%)	0.678
Chronic kidney diseases	4 (14.8%)	1 (10.0%)	3 (17.6%)	1.00
Clinical status at disease onset				
SOFA score	2.72 ± 1.78	1.67 ± 0.82	3.25 ± 1.91	0.073
White blood cell counts (blood test)	12.4 ± 8.19	14.0 ± 9.11	9.22 ± 5.25	0.256
C-reactive protein	9.30 ± 7.09	5.90 ± 2.55	11.0 ± 8.08	0.156
Pleural effusion test				
HA (× 10^3^ ng/mL)	26.5 ± 23.5	42.9 ± 23.3	16.8 ± 17.9	0.003
ADA (IU/L)	79.8 ± 74.3	89.7 ± 33.3	74.0 ± 90.9	0.032
LDH (× 10^2^ IU/L)	50.5 ± 128	8.46 ± 4.65	75.2 ± 157	0.195

Chronic respiratory diseases: COPD, bronchial asthma and interstitial pneumonia; chronic cardiovascular diseases: congestive heart failure and ventricular dysfunction; chronic kidney diseases: undergoing hemodialysis and after renal transplantation; HA: hyaluronic acid, ADA: adenosine deaminase, LDH: lactate dehydrogenase.

**Figure 1:**
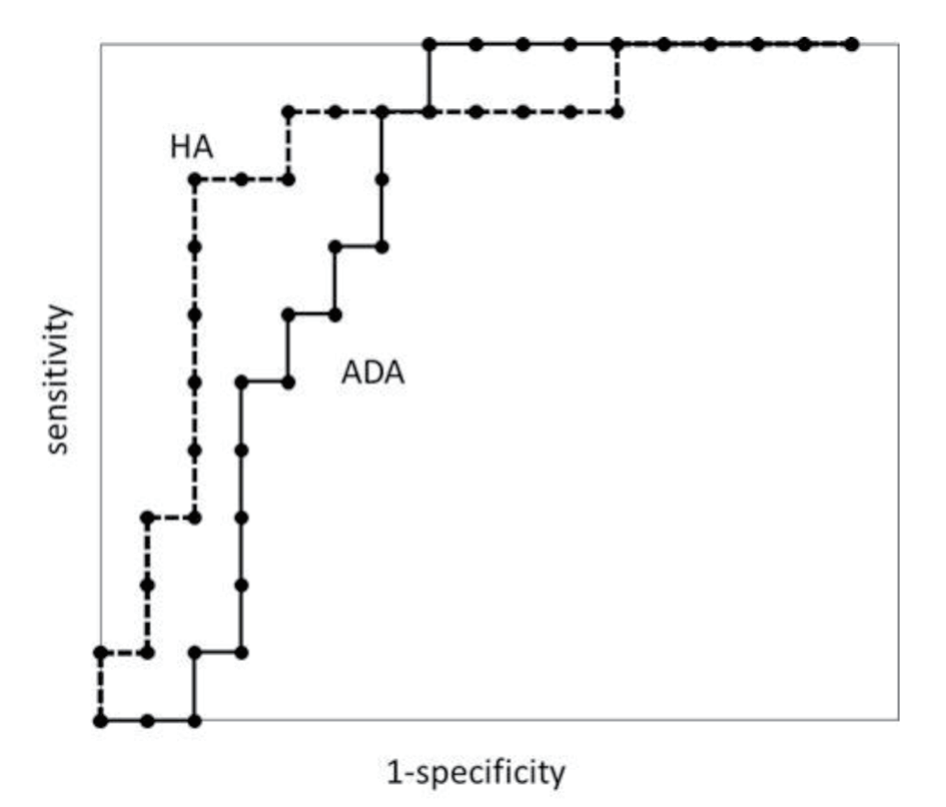
Receiver operating characteristic curves for pleural fluid adenosine deaminase (ADA) and hyaluronic acid (HA) in patients with tuberculous pleurisy.

There were no significant differences in AUC between the two groups (Fig. 1). The optimal cut-offs using the ROC curve were 24,950 ng/mL of hyaluronic acid and 51.9 IU/L of adenosine deaminase respectively (Table 2).

**Table 2:** The Optimal Cut-Off Level for Hyaluronic Acid and Adenosine Deaminase Concentrations in the Pleural Fluid of Patients With Tuberculous Pleurisy

	Cut-off	Sensitivity	Specificity	PPV	NPV	Likelihood ratio	Odds ratio
HA	24,950 ng/mL	0.80000	0.88235	0.80000	0.88235	6.8	30.0
ADA	51.9 IU/L	0.90000	0.64706	0.60000	0.91667	2.55	16.5

HA: hyaluronic acid; ADA: adenosine deaminase; PPV: positive predictive value; NPV: negative predictive value.

## Discussion

Our study results showed that hyaluronic acid concentration in pleural fluid was significantly increased in patients with tuberculous pleurisy, and that the use of this measurement was non-inferior to that of adenosine deaminase concentration in pleural fluid for the diagnosis of tuberculous pleurisy.

Hyaluronic acid can be produced from alveolar epithelial cells or mesothelial cells following local inflammation [6]. Indeed, a previous report showed that a high concentration of hyaluronic acid was seen in benign inflammatory diseases [2], particularly in those with long-term inflammation such as rheumatoid arthritis and complicated parapneumonic effusion [4]. Normally, tuberculous pleurisy presents with relatively few symptoms; moreover, and there is chronic inflammation [7]. This chronic inflammation may increase the hyaluronic acid concentration in pleural fluid. However, it was extremely difficult to calculate the duration of the inflammation.

Some reports have indicated that the production of hyaluronic acid might be promoted by proinflammatory cytokines such as tumor necrosis factor alpha and interleukin 1 beta [8, 9]. Thus, elevation of these cytokines in the pleural fluid can lead to increased hyaluronic acid concentration in pleural fluid. For example, in patients with complicated parapneumonic effusions, these two cytokines were significantly elevated in pleural fluid as was hyaluronic acid concentration [4]. Tahhan et al also showed that tumor necrosis factor alpha tended to be elevated in cases with tuberculous pleurisy [10], which is probably why, in our study, patients with tuberculous pleurisy had high hyaluronic acid concentrations in pleural fluid. Tahhan et al additionally suggested that tumor necrosis factor alpha level could be used to diagnose tuberculous pleurisy.

Diagnosis of tuberculous pleurisy using pleural fluid is difficult because the positive culture rate of *Mycobacterium tuberculosis *in pleural fluid is relatively low [5], often necessitating a pleural biopsy. In Japan, however, *M. tuberculosis* infection is often found in the elderly, in whom performing invasive procedures such as pleural biopsy is difficult; consequently, the measurement of adenosine deaminase concentration in pleural fluid has become a valuable diagnostic tool. Furthermore, some reports have shown that infective pleurisy not associated with tuberculosis, e.g., cryptococcosis and brucellosis pleurisy, could also result in a high concentration of adenosine deaminase in pleural fluid [11, 12]. Therefore, measuring hyaluronic acid in combination with adenosine deaminase in pleural fluid could lead to a more effective diagnosis for tuberculous pleurisy.

The present study has several limitations. First, it was retrospective, single-center study. Second, the sample size was small, because only hospitalized adult patients with infective pleurisy who met the criteria were enrolled. However, the study results demonstrate for the first time that the measurement of hyaluronic acid concentration in pleural fluid might be a diagnostic aid for tuberculous pleurisy. Nevertheless, a larger-scale prospective study is required to confirm our findings.
